# Two modes of being together: The levels of intersubjectivity and human relatedness in neuroscience and psychoanalytic thinking

**DOI:** 10.3389/fnhum.2022.981366

**Published:** 2022-09-08

**Authors:** Riccardo Williams, Cristina Trentini

**Affiliations:** Department of Dynamic and Clinical Psychology, and Health Studies, Sapienza University of Rome, Rome, Italy

**Keywords:** contingent intersubjectivity, mentalization, self-other representations, psychoanalysis, neuroscience, mirror neurons, affective resonance

## Abstract

The notion of intersubjectivity has achieved a primary status in contemporary psychoanalytic debate, stimulating new theoretical proposals as well as controversies. This paper presents an overview of the main contributions on inter-subjectivity in the field of neurosciences. In humans as well as—probably—in other species, the ability for emotional resonance is guaranteed early in development. Based on this capacity, a primary sense of connectedness is established that can be defined inter-subjective in that it entails sharing affective states and intentions with caregivers. We propose to define such a form of inter-subjectivity as *contingent*, since the infant’s early abilities for resonance do not imply the more generalized capacity to permanently conceive of the relationship outside the realm of current interactions and the infant-caregiver’s mutual correspondence of internal states. This form of connection, hence, results in a self-referential, bodily, and affectively codified, context- and time dependent, like-me experience of interactions. The gradual maturation of brain structures and processes as well as interactive experiences allow proper intersubjectivity exchanges, grounded on new intentional and representational capacities, to evolve. In this more mature form of intersubjectivity, the individual is allowed to conceive of her own psychic space both as distinct and as possibly connected with the other’s contents and experience, even in the absence of current behavioral indicators of such correspondence. This multi-layered model of intersubjectivity, which is embraced by current neuroscience research, seems to allow for new interpretations of psychoanalytic models of human relatedness based upon classic clinical observations.

## Introduction

The recent main theoretical and clinical innovations in psychoanalysis stem from a view that is based on the notion of primary intersubjectivity (Stern, 2005). The psychoanalytic bi-personal model conceives of the psychic as the product of the interiorization of real early interpersonal exchanges and human motivation for relatedness (Eagle, 2011). This new perspective has also led to previous psychoanalytic clinical theories models—founded on developmental notions, such as the phase of primary narcissism (Freud, 1914), normal autism and early symbiosis (Mahler et al., 1975), primary self-other undifferentiation (Winnicott, 1945; Jacobson, 1964; Kohut, 1971), and primacy of phylogenetically inborn phantasies that shape early representations of interactions (Klein, 1946; Kernberg, 1985)—to be questioned.

The current intersubjective or relational perspective heavily relies on infant research evidence (Beebe and Lachmann, 2002), claiming to abandon the so-called adult-morphic and pathomorphic psychoanalytical reconstructions of early infant development (Peterfreund, 1978). However, among developmental researchers, a consensus regarding the true intersubjective nature of early interactive experiences (Carruthers and Smith, 1996) remains far from being reached. Two main perspectives struggle in this area. According to the simulationist point of view, the early capacity to understand the other’s intentions and experience sharing is warranted by early mechanisms of imitation that allow the other’s intentions to be internally reproduced and matched with the observer’s own experience and intentions (Goldman, 2006). In the alternative view (the mentalistic perspective), no experience of sharing intentions can be achieved before the capacity for attributing mental states to the self and the other is established (Tomasello, 1999).

In this paper, we scrutinize the contributions of neuroscience in modeling the experience of interactions during development. To discuss the data from this field, we consider some basic prerequisites for the recognition of intersubjective capacity that are usually considered in developmental research, such as the infants’ ability to: a) form self-other unified representations; b) understand the intentions pertaining to self and others’ behaviors; c) establish a self-other differentiation; and d) be aware and understand that their own behaviors and those of others have the same intentions.

## A neuroscence perspective of intersubjectivity

### The bodily multimodal nature of early self and other’s representations

The bodily multimodal nature of early self and other’s representations has been widely considered in neuroscience literature. A recent perspective comes from Atzil et al. (2018), who propose that representations of social objects are built upon the regular association between interoceptive information about allostasis and exteroceptive information deriving from exchanges with the caregivers. Such a perspective introduces a more socially-oriented interpretation of the original definition of “allostasis,” according to which the brain is constantly engaged in regulating the organism’s internal milieu by anticipating needs and preparing to fulfill them before they arise (Sterling, 2012; Sterling and Laughlin, 2015). Atzil et al. (2018) claim that there is no “core social knowledge” at birth (Carey and Spelke, 1996; Spelke and Kinzler, 2007): this form of knowledge rather arises from the gradual learning about social agents and social behaviors. Since the brain “categorizes sensory information to predict about allostasis (…), sensory regularities (such as a face) will [thus] become concepts more rapidly if they impact allostasis. The association between allostasis and a human agent [such as the mother] will result in learning an important social concept: ‘mommy”’ (Atzil et al., 2018; p. 630). This (in our opinion, constructivist) model postulates that rudimentary social concepts develop in early infancy in the form of multimodal representations (such as maternal face) and become more abstract with development. Moreover, this model postulates that, through social regulation of allostasis, infants also acquire social competencies—such as synchrony— that they learn to intentionally use to regulate both their own and others’ allostasis (Atzil et al., 2011, 2018).

Indeed, supporters of primary intersubjectivity also believe that the capacity to build representations of the self and the other are based on an intermodal code. At the same time—assuming a very different perspective from that presented above—, they also believe that, already at birth, infants are aware of the presence of the other persons and (by virtue of that) can build representations of self-other interactions in the domain of actual experiences with others (Trevarthen, 1974, 1979, 1993, 1998).

The initial behavioral indicator of early intersubjectivity is represented by infants’ ability to imitate facial movements performed by an adult (such as the opening of the mouth and the protrusion of the tongue or lips), as documented by Meltzoff and Moore (1997) in their laboratory study on six newborns (of whom one was only 60 min old). Research has proved that imitation is not an automatic and involuntary reflex-like phenomenon, but a behavior toward which newborns are strongly motivated. Newborns, not only imitated gestures, but they also spontaneously “provoke” previously imitated gestures, waiting for the other to respond (Nagy and Molnar, 2004). Moreover, newborns can correct their own movements to make them converge with those of the observed adult (Meltzoff and Moore, 1997), and can reproduce gestures after a 24-h delay, from at least 6 weeks of age (Meltzoff and Moore, 1992; 1994). In this perspective imitative behaviors are interpreted as evidence of a precocious sense of self as differentiated from others and agent in the environment (Rochat and Striano, 2000). As Metzoff and Moore (1977) have stated, during imitation, the infant compares “the sensory information from his own unseen motor behavior to a ‘supramodal’ representation of the visually perceived gesture and construct the match required (…). [Imitative behaviors] are (…) accomplished through an active matching process and mediated by an abstract representational system” (p. 78). This “active intermodal mapping” (Meltzoff and Moore, 1997) is possible because the perception and production of self and others’ actions are represented within a common framework. Hence, in this stage, the infant “feels *what* the other feels.”

Numerous studies have provided the evidence that imitative capability is ensured by neural mirror mechanisms, allowing a shared mapping (and thus a common framework) between self and others, at the bodily level. Mirror neurons (MNs)—discovered in the ventral premotor cortex (vPMC) and the inferior parietal lobe (IPL) of macaque monkeys (Gallese et al., 1996; Rizzolatti et al., 1996)—are a distinct class of neurons that discharge when an individual performs a goal-directed action or observes someone else performing the same action. MNs—and the non-conscious, prereflective, and presymbolic functional mechanism that they underpin [i.e., the *embodied simulation* (Gallese, 2003)]—allow individuals who are confronting others’ behaviors to experience a specific phenomenal state of “intentional attunement” (Gallese, 2006). Such a condition generates a peculiar quality of familiarity with other individuals that is produced by the resonance of their emotions and intentions with the observer’s simulation.

Recent electroencephalography (EEG) studies have shown that shared representations between self and others are reflected by mirror mechanisms in the infant sensorimotor cortex (Simpson et al., 2014; Southgate et al., 2009, 2010), similar to those that are found in adult brains (Gallese, 2014). Notably, positron emission tomography (PET) studies have documented that metabolic activity is highest in the sensorimotor cortex, already before 5 weeks of age (Chugani, 1994; Chugani et al., 1987).

EEG studies on mirror mechanisms in infants have focused on the sensorimotor alpha (or mu) rhythm during action observation and action execution (Cuevas et al., 2014; Marshall et al., 2002; Marshall and Meltzoff, 2011). The mu rhythm is an EEG oscillation in the alpha frequency range (recorded over the central electrodes) that is generated in the resting state and desynchronized (i.e., attenuated or suppressed) prior to or during motor events. In infants, the observation of an experimenter who is performing a goal-directed action using a particular body part (hands or feet) is associated with desynchronization of the mu rhythm of the corresponding area of the body in the infant sensorimotor cortex (Marshall and Meltzoff, 2014; Saby et al., 2013). Similar somatotopic patterns have also been observed when infants perform the same actions as an experimenter (Marshall et al., 2013). Such somatotopic organization is considered as an index of the “intercorporeal mapping of corresponding body parts between self and other” (Marshall et al., 2013; p. 22), which allows an infant to engage in early imitation (Meltzoff, 1988; Meltzoff and Moore, 1997). It may be suggested that sensorimotor cortex guarantees the “supramodal” mechanisms (Meltzoff and Moore, 1997) that allow the infant to share the sensory feedbacks coming from the caregiver’s behaviors and those evoked by his own movements (Meltzoff and Marshall, 2018). Furthermore, data somatotopic organization may be considered by supporters of primary intersubjectivity as neuroscientific evidence of the “like me” simulationist framework (Meltzoff, 2007, 2013), according to which infants can parse a similarity (or equivalence) between their own bodily acts and those of others.

In a critical approach to the perspective of primary intersubjectivity, whether imitative behaviors clearly reflect the primary capacity to *understand* another’s intentions is questioned. Similar forms of imitations exist in other species (such as macaques), in which forms of proper intersubjective exchanges are not otherwise displayed (Gallese, 2014; Tomasello, 1999). Another critical point concerns the nature of the experiences that are ensured by early imitative behaviors. This issue has been discussed in the neuroscence literature, demonstrating that inner reproduction of another’s observed behavior is not followed by an *aware experience* of one’s own intention or that of others (Avenanti et al., 2005; Caramazza et al., 2014).

According to these criticisms, the low-level processing of observed actions in imitative behaviors does not suffice for an experience of intersubjective sharing which, instead, requires higher-level cognitive processes that allow intentional states to be inferred (Heyes and Catmur, 2022). As Di Bernardo (2021) has stated, “in order to establish full and effective emotional communication between two people, it is necessary for each of the people involved to let their own state of mind be influenced by that of the other, so that they ‘feel’ it and tune in to it” (p. 8). In response to these criticisms, simulationist theorists have focused on observational and neuroscience studies that appear to support the early achievement of intersubjective capacities.

### Understanding intentionality

After early imitations, other routines of mutually oriented interactions emerge gradually, including protoconversations (Trevarthen, 1998), affective tuning (Stern, 1985), and turn-taking behaviors (Weinberg and Tronick, 1997).

Neuroscience research has evidenced that these interactions are regulated by the right temporoparietal junction (rTPJ) (for a review, see Schore, 2021), an area located at the intersection of the posterior end of the superior temporal sulcus (STS), the inferior parietal cortex (IPC), and the lateral occipital cortex (lOC). Coherently, near-infrared spectroscopy (NIRS) studies on young infants have documented that activity in rTPJ is enhanced in response to social signals (such as human voice; Grossmann et al., 2010) and reciprocal interactions (Hakuno et al., 2020). Notably, the right lateralization of this cerebral system is consistent with Schore’s (2021) perspective on interpersonal neurobiology of intersubjectivity and with what Decety and Chaminade (2003) have reported in the conclusions of their review: “intersubjective processes are largely dependent upon the right hemisphere resources, which are the first to develops” (p. 591).

In these types of interactions, infants show the capacity to modulate their behaviors by anticipating another’s intention, to reach a condition of sharing their affective experience (Stern, 1985). The experience of intersubjective sharing necessarily implies the capacity to connect one’s own intentions to the other’s intentions: therefore, it has as its prerequisite the capacity to understand intentions.

Current empirical and theoretical orientations conceive intentionality not as a unitary capacity, but as a hierarchy of abilities (Bekoff, 2007; Dennett, 1991; Tomasello and Call, 1997). At the first level (which is shared by many species), intentionality implies the capacity to have a belief about an object. The second level of intentionality entails the capacity to have a belief about another agent’s belief and regulate one’s behavior consequently. Infant observation has provided evidence for the presence early in infancy of this level of intentionality and, by virtue of that, of primary forms of intersubjective sharing. This second level does not require the capacity to explicitly represent believes as such and can be observed in many species, including animals’ deceitful and playful behaviors (Tomasello et al., 2003; Jamieson and Bekoff, 1996). The third level of intentionality (which is instead most typical of human beings) is characterized by a reflective form of intentional attributions, in which an individual is able not only to have a belief about another individual’s belief but also to see whether the other’s belief corresponds or not to actual reality (Dennett, 1991). This higher form of intentional stance is linked to a more basic capacity to explicitly attribute and reflect upon mental states (Tomasello, 1999).

MNs are considered to provide a neuroscientifically viable account of intentional understanding (Gallese, 2014). The distinctive characteristic of MNs is that observing a motor act activates the same motor cerebral areas that are required by the observer to execute that same action (Rizzolatti and Sinigaglia, 2008). In line with this, the observer can directly understand others’ actions (the *what*) and to ascribe them intentions (the *why*), without the mediation of any cognitive or inferential processes (Rizzolatti and Sinigaglia, 2007; Rizzolatti and Fogassi, 2014). In recent years, the MNs perspective has proposed an innovative interpretation of intention understanding and early forms of sharing. As stated by Gallese (2014), the intersubjective connection that is allowed by MNs at early stage of development remains at a “subpersonal” level and do not fully account for the complexity of interpersonal experiences that occur in later stages of development, although they have to be regarded as the building blocks of any more complex intersubjective experience in human species. It has been stressed that the understanding and sharing of intentions is codified “via a mechanism of action representation [in which the insula plays a fundamental role] shaping emotional content, such that we ground our empathic resonance in the experience of our acting body and the emotions associated with specific movements” (Carr et al., 2003; p. 5502). It follows that this type of intersubjective connection is limited to the sensory-motor repertoire that is available to the observer (Buccino et al., 2004; Simpson et al., 2014)—i.e., the infant’s activation of single schemes of movements—, and that does not fully replicate the complexity of a caregiver’s behaviors.

Further, the experience of “being together” (Stern, 2004) is circumscribed to the duration of the ongoing interaction with the caregiver and is conceived as occurring at a sub-personal, pre-reflective level, where a sense of being a self in the relationship with another self is not yet developed. Given these features of the experience of early intentional understanding, we propose defining this first level of sharing as “contingent intersubjectivity.”

In the current debate on primary intersubjectivity, there is another key controversy to be dealt with.

### Self-other differentiation

For of the experience of *sharing* to occur, an infant must be able to achieve a sense of “ownership” of personal experience that allows her to differentiate her own from other’s experience (Gallagher, 2000): and thus, “to feel *that* the other feels.” Without this capacity, the sharing of experience warranted by the MNs simulation model has to be meant as a source of emotional contagion (de Waal, 1996). To understand the birth of the ability to create the experience of ownership and the self-other differentiation, current neuroscience research has proposed a cogent model of the development of the self.

Recent contributions conceive the self as a multilayered construct which originates in the course of the development, drawing on different sources of internal and external information, and also recruiting diverse brain structure into a progressively integrated complex neural network (Northoff and Scalabrini, 2021; Qin et al., 2020). Assuming this perspective, the large-scale meta-analysis on neuroimaging studies by Qin et al. (2020) proposes a nested hierarchy model of self which includes three intimately connected layers of processing: interoceptive-processing, exteroceptive-processing, and mental-self-processing. This model implies that cerebral regions of the lower level are included in the next higher level and then implemented with additional regions. Therefore, each of the hierarchical layers of self-processing recruits both overlapping and separate brain areas.

At a first level, the implicit (that is, not involving attention) interoceptive-processing is generated by the multimodal representation of the signals provided by the activation of the cardio-circulatory, respiratory, gastrointestinal, and urogenital apparatuses (Fotopoulou and Tsakiris, 2017; Tsakiris, 2017). The integration of such interoceptive signals is mediated by several brain areas, including the bilateral insula, dorsal anterior cingulate cortex (dACC), thalamus, and bilateral parahippocampus gyrus. This network is similar across all mammalian species and comprises mainly regions belonging to the salience network (Menon, 2011). The multimodal representation provided by the integration of interoceptive stimuli may be considered as the somatic marker that establishes the *proto-self* (Damasio, 2010), without which the more advanced aspects of the self could not take place (Gallagher, 2000).

The sense of the self—conceived of as both differentiated and in relation with other individuals—relies on the capacity to attribute the experience derived from internal processes (that is interoceptive, motor, emotional, and cognitive processes) to an objectified representation of the self. The sense of *ownership* characterizing the basic as well as the fully-developed (that is, reflective) forms of self-awareness is based upon this process of self-attribution. For many years, it has been proposed that the first form of objective self-representation was reached at a visual level and implied the capacity for the infant to recognize herself in the mirror, an ability appearing not before 16–18 months of age (Gallup, 1998). More recently, it has been suggested that a process of objective self-recognition can be operated earlier, drawing on other sources of exteroceptive information, such as visual, tactile, proprioceptive, and multi-sensory information (Gallagher, 2005). Assuming this perspective, the objective self-recognition gradually emerges in the course of development, involving (at early developmental stages) only singular aspects of the body scheme (e.g., face, hands) and motor programs (Fotopoulou and Tsakiris, 2017). In line with the model proposed by Atzil et al. (2018), the possibility to match interoceptive with exteroceptive information is thought to be allowed by the existence of Bayesian computational mechanisms that predict the frequency of the co-occurrence in the activation of these two types of information (Tsakiris, 2017).

In the hierarchy model proposed by Qin et al. (2020), the second layer (that is the exteroceptive-processing) not only integrates exteroceptive, proprioceptive, and multisensory proprioceptive inputs, but it also links the intero- and exteroceptive body signals with signals coming from external environment, modulating basic self-other boundaries (Park and Blanke, 2019; Suzuki et al., 2013; Tsakiris, 2017). These stimuli are processed by a cerebral network that includes the bilateral insula, anteromedial prefrontal cortex (amPFC), PMC, and bilateral TPJ. This layer also comprises regions typically involved in face-recognition (such as the right fusiform gyrus) and sensorimotor areas (such as the postcentral gyrus). Interestingly, Northoff and Scalabrini (2021) have suggested that “as these regions process inputs from different sensory modalities, they may be key in not only integrating extero- and proprioceptive modalities but also different exteroceptive sensory modalities, that is, cross-modal integration” (p. 7).

The third and highest level (that is the mental-self-processing) includes into the contents of the self also stimuli generated by higher order cognitive processes (rather than only body-based physical) stimuli (Qin et al., 2020). This layer recruits the bilateral insula, PMC, bilateral TPJ, and also crucial areas of the default-mode network (DMN)—such as the pregenual anterior cingulate cortex (pACC)/amPFC, posterior cingulate cortex (PCC)—that are implicated in self-referential processes.

Taken together, these data seem to show that any level of self-representation always implies the activation of those brain structures that are recruited at the level of the core interoceptive self-representation. This evidence has important consequences in that it shows that the contents of self-representations are unavoidably shaped by bodily experiences connected to physical needs and emotional activations, as otherwise stated by the psychoanalytic theories. Indeed, the activation of the insula may represent the somatic marker that enables to distinguish between what belongs to the self (because associated to bodily interoceptive information) from what has to be regarded as non-me. TPJ is implicated in high-order cognitive functions—such as theory of mind (TOM; Chan and Lavallee, 2015; Schurz et al., 2017) and perspective taking (Schurz et al., 2013)—that require a concomitant representation of both self and other (Schurz et al., 2013). By virtue of their anatomical connections (Saur et al., 2008), the TPJ and insula are thought to collaborate to provide the bodily self-consciousness (Park and Blanke, 2019), which “could serve as the basis for further co-representation of social information pertaining to both self and other, to enable efficient interactions with the external world” (Qin et al., 2020; p. 89).

Other authors have specifically investigated the question of self-other differentiation with regard to the domain of activation of MNs. Results from this research area seem to show a degree of convergence with the nested hierarchy model of self, proposed by Qin et al. (2020).

Several studies have mapped the activation of specific cerebral areas while subjects were executing, thinking of, or simply imaging a plan of action in the first or third person, finding all the experimental conditions activate areas with MMs. Moreover, when subjects were asked to observe or think of another agent’s finalized behavior without assuming the third-person perspective, the task automatically led to a default mode (DM) of self-attribution of the feelings, that were produced by the simulation (Decety and Ickes, 2009). The DM of self-attribution is associated with activation of the mPFC, pACC, PCC, and temporopolar cortex (TPC) (Davey et al., 2016; Dixon et al., 2022; Northoff et al., 2006); moreover, it is also associated with areas involved in the synthesis of proprioceptive inputs (such as the insula) that modulate the integration of the body scheme (Cabanis et al., 2013).

The DMN rules out the possibility of proper intersubjective sharing and is used to account for the experience of *emotional contagion* (de Waal, 1996), which has been observed in certain clinical conditions (such as autism and schizophrenia) following imitative behaviors (Singer and Frith, 2005). Conversely, tasks that involve imitation or attribution of intentions to another agent are linked to activation of the right inferior parietal lobule (IPL), which appears to mediate the basic distinction between actions that are generated by the self and those by another agent (Decety and Jackson, 2004).

According to the neuroscience literature (Decety and Ickes, 2009; Gallese, 2014), the activation of mirror mechanisms during imitation is accompanied by the capacity to distinguish the sources of internal and external agency as early as 3 months of age (Gallese, 2014). As discussed above, the possibility to establish a sense of sharing necessarily requires a self-other differentiation (not to be experienced as an affective contagion). This pre-condition relies on the sense of ownership that is carried out through the coupling of interoceptive experience to an at least partially integrated exteroceptive representation of the self (including proprioceptive feedback involved in the sense of personal agency). It still remains matter of debate and empirical research whether this objectified integrated self-representation can initially be brought about by the mere capacity to distinguish the sources of agency emerging at this stage (Marraffa and Meini, 2019; Gallese, 2014).

Also, mentalistc intersubjective exchange of later stages of development hinges on a sense of ownership of one’s intentional states, conveyed by a basic self-monitoring mechanism of agency and bodily activation (Cermolacce et al., 2007). This assumption is supported on several levels. The activation of specific cerebral areas that govern the attribution of the sources of an action to the self or to the other has been observed in tasks that involve direct imitation and in the imagining plans of action, thoughts and emotions, autobiographical memory, and attribution of personal pronouns (Decety and Ickes, 2009), and is reported to be inverted in certain subjects (such as those with schizophrenia), who show a deficit in holding a sense of ownership of their own actions and metal processes (Farrer et al., 2003).

### Understanding the other and the self as mental agents

As Fonagy et al. (2002) have argued, in everyday life, we take it for granted that in interpersonal relationships, “I keep in mind your mind and you keep in mind mine” (p.375). This understanding of relationships as *a meeting of mental states* allows humans to experience a sense of personal connection when the other is physically absent or when the other shows intentions and goals of action that differ from ours, in complementary or even contrasting ways. Further, this background of an interpersonal connection allows us to appreciate the continuity of our current experience of sharing against previous encounters with the same person, characterized by different affective tones and motivations. This understanding is what allows *contingent* intersubjective exchanges to become an experience of relatedness. The complex level of intersubjectivity—indicating that “I know that you know that I know”—conveys a sense of mutuality and personal recognition and can only be reached gradually during ontogenesis, typically distinguishing the human species (Tomasello, 2019). As reported, earlier forms of intentional attributions that are based on simulation are insufficient to justify this complexity, and other mental prerequisites must be achieved by the infant. Indeed, current research shows that the quality of contingent intersubjective exchanges does not predict the quality of later intentional attributions (Moll et al., 2021): the new level of mentalistic understanding should be supported by a wider capacity—i.e., TOM (Baron-Cohen, 1991) or reflective functioning (Fonagy et al., 2002).

Developmental research has shown that early intentional attributions are present in early infancy but that they are better described as a teleological stance in which the understanding of intentions is directly derived from the outcome of the agent’s behavior (Gergely and Csibra, 2003). To overcome this teleological thinking and to “decouple” internal states from outer reality, humans must become aware that actual behaviors are the consequence of an internal disposition that is conceived as intentions, thoughts, emotions, and desires. Developmental researchers have highlighted that such “intentional stance” or “second level intentionality” (Dennett, 1991) can only be reached through an objective representation or “second-order” representations of one’s own and others’ internal states (Fonagy et al., 2002). The first behavioral evidence of this developmental achievement is provided by the acquisition of an infant’s engagement in triadic (infant-other-object) joint attention interactions (Tomasello, 1995).

In human adults, joint attention skills are sustained by the dorsal region of the medial prefrontal cortex (mPFC) (Frith and Frith, 2006). Behavioral research has largely documented that infants are already able to discriminate between dyadic and triadic joint attention intercourses at age 3 months (Striano et al., 2005). Nevertheless, a full understanding of joint attention does not exist until age 9 months (Tomasello et al., 2005), perhaps by virtue of increased metabolic activity, which occurs in the frontal areas at approximately age 8 months (Chugani, 1994; Chugani et al., 1987).

EEG studies on the negative component (Nc) of event-related potentials (ERPs) support this assumption. The Nc is a negative deflection in frontocentral electrodes that is believed to reflect attentional orientation to salient stimuli (Richards et al., 2010; Striano et al., 2006) and attentional arousal (Soto-Icaza et al., 2015), because its amplitude is larger during sustained attention (Richards, 2003). In this domain, 9-month-old infants who engage in a joint attention interaction show higher amplitudes in the Nc of ERPs compared with non-joint attention intercourse (Striano et al., 2006).

According to the embodied simulation framework, ToM relies on the capacity to adopt a simulation routine that is, in turn, allowed by MNs (Gallese and Goldman, 1998). Nevertheless, as Frith and Frith (1999, 2001) have emphasized, the conscious reflection of one’s own mental states and those of others requires resources beyond the capacity to simulate or imitate an action and that are associated with the development of executive functions, especially inhibitory control (Carlson and Moses, 2001; Decety and Jackson, 2004).

Notably, the ToM reliably engages a network of brain regions that overlap partially with those that are involved in executive functions, including the mPFC, inferior frontal gyrus (IFG), IPL, and TPJ (Frith and Frith, 2012; Frolli et al., 2019; Gallagher and Frith, 2003; Koster-Hale and Saxe, 2013; Wade et al., 2018). Using near-infrared spectroscopy (fNIRS), Hyde et al. (2018) demonstrated that 7-month-old infants who stare at events evoking the ToM activate the TPJ but no other temporal regions (such as the superior temporal sulcus – STS) or the prefrontal regions (such as the mPFC). These data shed light on early organization of cerebral networks involved in the ToM, during infancy. At this early developmental stage, while the TPJ is already functionally organized for processing social stimuli that are relevant to the ToM, the mPFC (involved in inhibitory control; Draperi et al., 2022) might not be, due to its slow maturation during the first year of life (Chugani, 1994; Chugani et al., 1987).

## Summary

**FIGURE 1:**
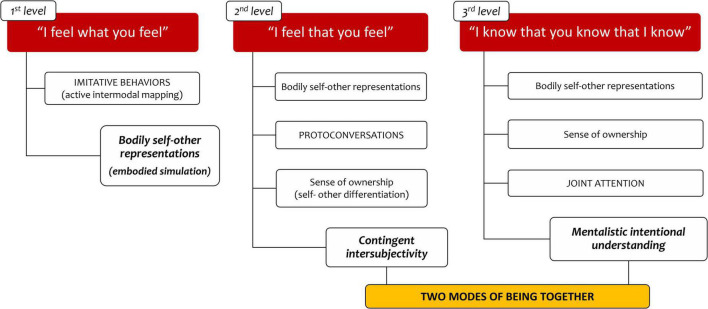
Two modes of being together model.

Current neuroscience research is detailing early abilities that support the development of intersubjectivity. A putative and provisional reconstruction of what the human experiences in early social interactions can be obtained, relying on a multidisciplinary perspective (see Figure 1).

It appears that, during the first year of life, an infant can have an experience of sharing with others, which we define as “contingent intersubjectivity.” This form of connectedness (which can also be postulated in other species) is supported by neural mirror mechanisms and self-monitoring processes that enable the infant to distinguish internal from external sources of experience and to develop a sense of self based upon ownership and agency (at least on the level of interoceptive, tactile and sensory-motor information). We contend that this level of sharing possesses the prerequisites of intentional understanding and can thus be considered as a primary form of intersubjectivity. However, this form of intersubjective sharing seems to have some peculiarities that distinguish it from more mature forms of intersubjectivity.

In the first place, contingent intersubjectivity is temporarily limited to the ongoing interactions. At this level, the quality of relational experience is totally shaped by the actual affective and communicative exchanges between the infant and her caregivers, and no integration of such singular experiences can be achieved to establish the stable sense of connectedness that characterize interpersonal relationships. Secondly, contingent forms of sharing can be achieved only through actual correspondences between the infant and the caregiver’s intentions. As reported in psychodynamic literature (Weinberg and Tronick, 1997), when no such intentional attunement is reached, the sense of affective connection tends to decline. Furthermore, contingent intersubjectivity is self-referential, in that the infant, by default, feels the quality of the affective experience that is shared with the other as being hers. This means that, when the other exhibits disruptive or intrusive communicative behaviors, the infant tends to attribute to herself the caregiver’s negative internal states (Fotopoulou and Tsakiris, 2017; Lyons-Ruth et al., 2006). Thirdly, and most importantly, the experiential contents of contingent intersubjectivity are not formulated in terms of mentalistic explicit contents such as believes, thoughts, desires, emotions, intentions or goals. More peculiarly, the experience of contingent sharing is codified as a unitary form of experience, modeled by different sources of bodily and emotional information. Finally, it has to be specified that the lack of the capacity for mentalization of such experiences of self-other interactions differentiates the basic form of intersubjective sharing from proper bi-personal connections, which are instead characteristic of more mature forms of human relatedness.

### Implications for the dialog between psychoanalysis and neuroscience

We would like to stress that the reasoning we propose in this paper is also aimed at confronting two apparently incompatible ways of interpreting intersubjectivity, which is, the embodied simulation perspective and the mentalistic perspective. Specifically, our discussion highlighted that all experiences of intersubjective sharing are anchored to a bodily representation of interactions that comes into play both as a marker of self-other distinction and as a metaphorical bridge to enter and model the other’s intentions and experience. As discussed above, the acquisition of the ability for mind-reading affords the individual a new and more ample way of experiencing interactions that is based on the capacity to live the current interactive exchanges as only one of the possible experiences of the relationship. We believe that these two tenets of our discussion bear some important theoretical and research consequences for the dialog between psychoanalysis and neuroscience.

One important aspect that needs to be clarified in future studies is the influence of early experiences of intersubjective sharing on the development of the mentalized forms of relatedness. Research shows that impairments in the bodily self-monitoring may undermine reflective self-other representations (Tsakiris, 2017). At the same time, no clear continuity can be established between the quality of early intersubjective experience and future metalizing capacities (Moll et al., 2021).

Far from falling back upon a reductionist perspective, current neuroscience research has identified the multifaceted nature of mental life, providing a new and articulated perspective on the relationships between the multifaceted aspects of the individual experience of the self in relation to her social environment. The contributions included in this paper clearly evidence the bodily foundation of human psychic life, as affirmed by the psychoanalytic thinking from its very beginning (Freud, 1914; Heimann et al., 1952). At the same time, psychoanalysis has always dealt with the necessity to understand the psychic principles that lead the transformation of the unaware experience deriving from bodily functioning into conscious thoughts and emotional contents (Bion, 1962). Therefore, for both researchers and psychoanalysts, an important area of investigation definitively concerns the kind of mutual influence between the representational codes and the quality of experience pertaining to the contingent and reflective modes of intersubjective experiences. Investigations into the neural networks supporting each aspect of emotional sharing indeed point to the issue of how the brain functioning reaches the integration between bottom-up and top-down processing of self and relationships, leading to personal meaning (Northoff and Scalabrini, 2021)

We believe that a new important step for psychoanalytic investigations—as led by neuro-psychoanalisys and neuroscience—has already been achieved. The affirmation of bi-personal models in current psychoanalytic thinking (as opposed to the mono-personal framework of classic psychoanalysis) has correctly highlighted the importance of real social experiences for the development of personality and psychopathology (Gill, 1994). Current psychoanalytic relational orientation as well as infant research and attachment theory (Bowlby, 1969/1982) posit that the human fundamental motivation relies in the seek to establish a harmonic relationship with the caregivers, who are to meet this relational trust by offering recognition, regulation, sharing, and protection to the infant’s biological and emotional needs (Eagle, 2011). Coherently, it is also postulated that the positive experience of early relationships *de facto* entails a natural equivalence between the individual’s needs, her affective experience, and the object’s presence. It seems that such a polarized view runs the risk of throwing away the psychoanalytic baby with the bathwater.

Current neuroscience research seems to afford a more complex view of human relatedness. The presumed infant’s capacity to realistically appreciate and experience interpersonal relationships as such seems indeed the result of a progressive construction, depending on both maturation and experience. Early forms of relatedness are bound to a bodily experience constituting the idiosyncratic and personal meaning that shapes individual sense of the self in relation to the other (Modell, 1993). It should not be underestimated that psychoanalytic observations have deserved recognitions for having connected psychopathological states to the effects produced by the incompatibility between the underworld of private representations and the more mature and articulated experiences of relationships.

### Possible implications for psychoanalytic theory and clinical models

In this regard, we contend that the multi-layered account of the development of intersubjective capacities might help modeling some of the clinical phenomena envisioned by classic psychoanalysis, in an updated theoretical framework.

By virtue of the acquisition of mentalistic abilities, the creation of a virtual internal space that belongs to the self and the other allows the individual to experience that she and the other have different feelings and thoughts about the world, and that they can come to share a unique experience or re-establish a connection when this connection is lost. These achievements allow the individual to stay within and without the current experience of the self and the other. Notably, neuroscience research also shows that this developmental conquest does not rule out the embodied knowledge mechanisms that allow for primary forms of sharing and perceptions of another individual’s intentions. Neural mirror mechanisms, along with self-monitoring of bodily activation occurring in the interaction, constitute the basis for self-other experience, representing a “metaphorical bridge” for interpreting the meaning of ongoing relationships throughout life (Modell, 2003).

We suggest that our theoretical proposal might also shed a new light on some classic psychoanalytic observations of clinical phenomena observed, for instance, in borderline conditions. The “lack of constancy of the libidinal objects” (Mahler et al., 1975) and the ensuing fears of abandonment and symbiotic swallowing characterizing these patients may be read as the difficulty to integrate the contingent intersubjective experience into a mentalistic understanding of relationships. On the one hand, the fall in the mentalizing abilities may lead these patients to experience the absence of any contingent response on the part of the other as a definitive loss of the relationship, generating a state of extreme and hopeless solitude. Moreover, the ensuing strong thrust to regain a sense of relationship may lead these patients to evoke forms of contingent exchanges with the other, in which past or present experiences of rejection, frustration or intrusion may take over. The self-referential and all-encompassing mode accompanying contingent exchanges may plunge these patients in a state of self-other confusion, in which the sense of personal identity is lost. In a similar vein, it has been suggested that the feeling of entrapment into the repetition of a “traumatic script” (Meares, 2012), the identification with the traumatic object, and the difficulty to disentangle the self-experience from that of the object showed by borderline patients (Gabbard et al., 2006) may be related to the activation of memories of contingent interactions with the traumatizing figures, that are not sustained by an adequate cortical activity of second-order representations (Fonagy et al., 2002).

We would finally like to stress how the multi-layered view of intersubjective experiences emerging from the neurosciece and neuropsychoanalytic investigations bears some important consequences for the conception of treatment. Some recent psychoanalytic orientations (Bateman and Fonagy, 2013) have emphasized the importance to help the patient to mentalize her dispositional states to achieve a good therapeutic outcome. Undoubtedly, enhancing the patient’s reflective functioning is the unavoidable tool of work for any psychodynamically inspired psychotherapy. Nevertheless, the perspective presented in this contribution also points to the necessity to bring reflection into the field of the embodied experience of relationship. The creation of a new awareness of oneself should not be thought of as an epistemological effort (Ogden, 2019). Rather, reflection and interpretation should directly address those idiosyncratic bodily experiences of the relationship that constitute the building block and raw material upon which the sense of being a person in relation to the world comes to life and must be constantly re-created.

## Conclusion

In this paper, it has been proposed that neuroscience and psychoanalysis now converge in showing that there are at least two modes of being together. The multi-colored emotions and perceptions we live from within our body in the encounter with the others is what tells us how it feels to be in a relationship and to live a life. At the same time, we must or should be able to see and think about ourselves from without, to reach and maintain the sense of being a person, no matter what the ongoing interaction is. The contingent mode of being together provides the emotions and sensations that render the relationships meaningful and worth-living, while the reflective mode of being together allows us to create a history of these meaningful relationship, for good and for bad. There is always price that we pay to live relationships from both perspectives. It is the possibility to oscillate and compound these two modes of being together that makes us humans.

## Author contributions

RW conceived the work. Both authors searched and studied the literature, wrote the article, and contributed to the viewpoint that the article expresses.
